# Improving women’s experiences with gestational diabetes from culturally and linguistically diverse backgrounds in Australia: a qualitative study

**DOI:** 10.3389/fpubh.2023.1291347

**Published:** 2024-01-05

**Authors:** Hiu Wing Rachel Lau, Johnathon Dong, Tessa Weir, Meenakshi Chopra, Lyn Olivetti, Gregory Fulcher, Sarah Glastras

**Affiliations:** ^1^Sydney Medical School, University of Sydney, Camperdown, NSW, Australia; ^2^North Sydney Endocrine Centre, Royal North Shore Hospital, St Leonards, NSW, Australia; ^3^Endocrinology Outpatient Clinic Service, Nepean Blue Mountains Hospital, Penrith, NSW, Australia; ^4^Integrated Digital Enablement Accelerator (IDEA) Team, NSW Ministry of Health, St Leonards, NSW, Australia; ^5^Northern Sydney Local Health District, St Leonards, NSW, Australia

**Keywords:** antenatal care, gestational diabetes, endocrinology, obstetrics, delivery of health care, patient experience

## Abstract

**Introduction:**

Gestational diabetes mellitus (GDM) is the fastest growing type of diabetes in many countries worldwide, including Australia. Although studies have explored the experiences of women with GDM from ethnic minority groups, few have compared their experiences with women from Anglosphere backgrounds.

**Objective:**

To investigate the responses to diagnosis, the management of GDM, and the experiences of healthcare services among women with GDM from different culturally and linguistically diverse (CALD) backgrounds.

**Methods:**

Participants were recruited via convenience sampling by advertisement posted around antenatal clinics of three hospitals in NSLHD: Royal North Shore, Hornsby, and Manly Hospitals. The interviews were semi-structured, one-on-one, and in-person conducted by a trained female volunteer. The interviews were audio-recorded, transcribed into text. The data was analyzed via an inductive and descriptive coding approach. The codes were then categorized into main themes and sub-themes.

**Results:**

30 women (7 Australian-born, 11 Chinese, 8 Indians, and 4 Koreans) partook the semi-structured interviews and 5 themes were identified: (1) Reaction to diagnosis; (2) Management issues; (3) Roles of friends and family; (4) Information access; and (5) Experience with healthcare services. The lack of culturally tailored dietary information, social support and language barriers were the main factors underpinning the differences in GDM experiences among women from CALD backgrounds versus Australian-born.

**Conclusion:**

Healthcare models should provide more emotional support upon diagnosis, culturally tailored guidelines for lifestyle modifications, and involve friends and family in care and management to enhance the experience of GDM for women from CALD backgrounds.

## 1 Introduction

Gestational diabetes mellitus (GDM) is the fastest growing type of diabetes in many countries worldwide, including Australia (1). In 2014, the Australasian Diabetes in Pregnancy Society (ADIPS) adopted the new International Association of the Diabetes and Pregnancy Study Group (IADPSG) criteria for the diagnosis of GDM (2). This decision was based on the Hyperglycaemia and Pregnancy Outcome (HAPO) Study that demonstrated an association between adverse pregnancy outcomes, such as risk of developing type 2 diabetes after pregnancy or later in life, and increasing blood glucose levels below the old diagnostic criteria (3). Hyperglycemia is also associated with increased risks of adverse infant outcomes, including macrosomia, excess infant adiposity, and neonatal hyperinsulinemia (4). The new diagnostic criteria may increase the prevalence of women diagnosed with GDM in many countries, including Australia (5, 6).

Ethnicity has been identified as a risk factor for developing hyperglycaemia in pregnancy (7, 8). A study conducted in Victoria, Australia found that while almost 25% of women giving birth were born outside of Australia, this group of women constituted almost 41% of GDM cases (7). In particular, Asian women accounted for the highest incidence of 11.5% compared to those who were born in Australian at 3.7% (7). Increased ethnic diversity in countries, such as Australia, is considered a contributing factor to the increasing trend of GDM incidence (7, 8). In addition to the fact that the incidence of GDM is rising, there is a greater need to provide optimal care and management for Asian women.

Multiple studies have investigated the impact of GDM on women. Upon diagnosis of GDM, women might feel distressed, overwhelmed, shameful, guilty, etc. (9). Women with GDM had mixed experience in quality of care and had varying levels of knowledge regarding GDM (9). Stress related to dietary modifications were common among women, particularly women from culturally and linguistically diverse (CALD) backgrounds (9–11). Women from CALD backgrounds might also have difficulty practicing family traditions or cultural practices due to dietary restrictions (10, 11).

Although studies have explored the experiences of women with GDM from ethnic minority groups, few have compared their experiences with women with ethnicity from Anglosphere backgrounds or those identify as Caucasians (11, 12). The objective of this study was to identify the issues experienced by women with GDM from CALD backgrounds regarding its diagnosis, management, and their experiences with health services compared to Australian-born women. The secondary objective was to identify gaps in healthcare systems to improve the care provided to women with GDM from CALD backgrounds.

## 2 Materials and methods

### 2.1 Study design and participants

China, India, Korea and Hong Kong were the most common non-English speaking countries residing in Northern Sydney Local Health District (NSLHD) (13). Women were eligible to participate in the study if they were > 18 years old, diagnosed with GDM at least 6 weeks ago at the time of interview, and were born from 3 non-English speaking countries in the NSLHD (i.e., Chinese, Indian, Korean) or they were born in Australia and identified as Australian or Caucasian. Country of birth was identified from the Obstetrix database (an NSW-state based obstetric database), and the participants confirmed that their ethnicity was congruent with their country of birth. Study exclusions included diagnosis of diabetes before pregnancy, multiple pregnancies, and medical, obstetric, or foetal complications at the time of interview.

An qualitative methodology was chosen for this study as it would provide more depth and breadth into the experiences of women with GDM (14). It would also provide a greater understanding of patient’s beliefs and attitudes towards managing GDM and quality of healthcare they received, as well as how culture may play a role in their experience.

The interviews template was developed by authors MC, LO, GF, and SG. It consisted of open-ended questions regarding the self-management of GDM, lifestyle modifications (such as diet and physical activity), and experiences with health services. The interviews were semi-structured, one-on-one, and in-person conducted by a trained female volunteer, who was a midwife and was not involved in the study design, recruitment, or interpretation of outcomes. The interviews were conducted in a private room at the antenatal clinic. An interpreter, who provided in-hospital verbal and/or written translation services, was available during the interview upon request. If the participant felt upset or stressed during the interview, they were allowed to skip the question or stop the interview entirely. The interviewer would then provide counselling or other appropriate support for the participant.

The interviews were audio-recorded and then transcribed into text by author MC, who was a Multicultural Health Development Officer at the time of interview. Interviews were translated into text by interpreters if they were involved in the interviews. Neither the volunteer, the interpreters, nor the author MC were involved in the care and management of the participants to avoid bias in data analysis.

### 2.2 Data collection and analysis

This study was conducted between November 2016 and May 2017. Participants were recruited via convenience sampling by advertisement posted around antenatal clinics of three hospitals in NSLHD: Royal North Shore, Hornsby, and Manly Hospitals. Advertisement posters were also translated into Chinese and Korean for women from CALD backgrounds. It was not translated to the languages of India due to the diversity of dialects. All patients provided written consent to participate in the interview and for their data to be published in a peer-reviewed journal. An interpreter was present if requested or noted in the Obstetrix database to gain consent. Clinical staff involved in the care of participating women did not offer their patients to participate in the study. This was to minimize the influence of clinical staff on the participants’ response.

Transcripts were de-identified and uploaded to the NVivo software (v.1.5.1) for data analysis. The first author HL coded all transcripts with an inductive and descriptive coding approach (15). A preliminary thematic framework was reviewed by both author SG and HL. The codes were reviewed such that they appropriately reflected and summarized the content of the data. The codes were then categorized into themes by author HL, who also arranged the themes into a higher and lower levels. For instance, when asked about their reaction to their diagnosis of GDM, codes such as “upset,” “sad,” and “overwhelmed” were generated. These codes were subsequently grouped into the theme “emotional response.” This theme was then categorized into the main theme “reaction to diagnosis” along with “personal interpretation.” The final thematic framework was reviewed by author SG for consistency in interpretation. Common and contrasting data across different ethnicities were also identified within themes.

## 3 Results

### 3.1 Demographic characteristics

30 women (7 Australian-born, 11 Chinese, 8 Indians, and 4 Koreans) participated in the interviews. Interviews were conducted until data saturation was achieved, i.e., no more new themes emerged for the overall sample. The mean age was 33.0 ± 4.0 years, duration of the interview was 33.1 ± 14.6 min, mean maternal age, i.e., the age that the women were in their most recent pregnancy, was 33.6 ± 1.9 years, and median gestational age at diagnosis of GDM was 28.0 ± 4.2 weeks. Women from CALD backgrounds resided in Australia for a median of 5.2 ± 5.3 years. 2 participants required an interpreter (1 Chinese, 1 Korean). The details of the demographic characteristics of the participants are available in Table 1.

**Table 1:** Demographics of participants.

Characteristics	Number of participants *n* (%)
**Country of birth**	
Australia	7 (23)
China	11 (37)
India	8 (27)
Korea	4 (13)
**Age**	
25–30	8 (27)
31–35	13 (43)
36–40	9 (30)
**Weeks of gestation at time of interview**	
≤29	1 (3)
30–32	8 (27)
33–35	16 (53)
36–38	5 (17)
**Weeks of gestation at diagnosis of GDM**	
≤23	6 (20)
24–28	20 (67)
≥29	4 (13)
**Previous pregnancies**	
0	15 (50)
1	14 (47)
2	1 (3)
**Previous diagnosis of gestational diabetes**	
Yes	5 (17)
No	25 (83)
**Family history of diabetes**	
Yes	17 (47)
No	13 (43)
**Highest educational level**	
High school	2 (7)
Diploma	1 (3)
Bachelors	14 (47)
Masters	11 (37)
PhD	2 (7)
**Employment**	
Yes	24 (80)
No	6 (20)
**Number of years living in Australia** ^a^	
<5	12 (52)
6–10	6 (26)
11–15	3 (13)
≥16	2 (9)

a Only for women from CALD backgrounds (*n* = 23).

### 3.2 Themes and sub-themes

5 themes were identified from the interviews: (1) Reaction to diagnosis; (2) Management issues; (3) Roles of friends and family; (4) Information access; and (5) Experience with healthcare services. Their corresponding sub-themes were described as below.

#### 3.2.1 Reaction to diagnosis

##### 3.2.1.1 Emotional response

Most women, regardless of their ethnicity, felt surprised with their diagnosis of GDM (Table 2, Quote(Q) 1). Women from CALD backgrounds, particularly Indian and Korean participants, were more likely to feel distressed and overwhelmed with the diagnosis (Table 2, Q2). One Chinese women were not upset by the diagnosis as they perceived it to be common among pregnant women (Table 2, Q3).

**Table 2:** Themes and subthemes from the interviews.

Themes	Subthemes	Quotes
Reaction to diagnosis	Emotional response	*1. I was really quite surprised as I exercised heavily through the pregnancy and I eat quite well. I was not having to make many dietary changes and I was really surprised that I had it. (P10, Australian-born)* *2. I was crying for a couple of days … I (still) feel upset even now occasionally. (P24, Korean)* *3. I did not worry too much. … a lot of women would have this condition. (P19, Chinese)*
	Personal interpretation	*4. In most cases the diabetes goes away however due to my age and both my parents being diabetic I am concerned that the diabetes may not go away after the pregnancy. (P19, Chinese)* *5. … what is it (gestational diabetes) doing to the baby and especially when you think about Type 2 diabetes that they can get later in life. (P10, Australian-born)* *6. I have also liked sweet foods during this pregnancy. I usually do not like sweets but this pregnancy but this time like ice creams. It is really hard to control carbohydrates and I love carbohydrates. (P19, Chinese)*
Management issues		*7. With noodles I feel hungry very quickly. It is digested very quickly … I double the volume of vegetables or eat wholemeal bread … and meat. (P11, Chinese)* *8. I did not feel like eating and made me nervous about eating or being scared about eating carbohydrate or not eating at all. (P10, Australian-born)* *9. [I am] trying to do the right thing. But some meals I know that [I am] not eating all the meals according to the instructions and due to friends gathering and in Chinese New Year it is very hard to watch your diet. (P19, Chinese)* *10. It really very much hurts my fingers. (P30, Korean)* *11. With my work I forget to check it 2 h after my meal. So I do it at 1 h or 3 h as I am doing rehearsals and cannot stop to check. (P7, Australian-born)* *12. Unhappy about the needles as well and the result. I worry about it every time I test. If I exercise I feel more confident about my readings otherwise I worry about it. But if I am at work I am sitting down and I cannot exercise I worry. (P8, Korean)* *13. I have always been doing exercise and going to the gym till now and after pregnancy I have quit. Now can do 20–30 min exercise however it is the weather. (P22, Indian)* *14. I do walking after meals but not every day. I enjoy the exercise though. However it is challenging and it does not seem to contribute to controlling the sugar levels and does not impact on that. It is mainly diet and insulin. (P19, Chinese)*
Roles of friends and family	Assistants in management	*15. He (husband) has quit drinking Coke and he really likes that… He has changed his diet to match with me. (P22, Indian)* *16. I have not told my friends here but family yes and they are supportive. Family supports over the phone. My close and good friends are not here-they are back in India or somewhere else… (P22, Indian)*
	Need of support group	*17. [It is] hard to find [support] for migrants who have come here as compared to those in India… So I am looking for support from people who are on the same boat. (P22, Indian)*
Information access	Dietary information	*18. Given a lot of information but do not know about Indian food habits because we eat a lot of rice. (P16, Indian)*
	Online resources	*19. The women write down their experience [on the website], like what food to eat and what to cook and foods from (the) same culture. It is all very useful … We all know about this site, a lot of migrant women know about it and it has a lot of information including education, pregnancy. It is an online community. (P27, Chinese)*
Experience with healthcare services	Support from healthcare providers	*20. Information on how to control your diet, monitor sugar, exercise, how much fruit and how glucose index goes up. (P16, Indian)* *21. They follow-up with me about the blood sugar level… They do not leave you alone even at home check* via *email and* via *phone. (P21, Indian)*
	Consultations	*22. Every time [I] see a different doctor (obstetrician) or a midwife, never the same person who is seeing me … although been seen by same endocrinologists. (P19, Chinese)* *23. I spend AUD$30 per fortnight on parking. They give me 9 am appointment and takes me 45 min to get here and then 2.5 h waiting … sometimes not worth going to work after that. (P14, Australian-born)* *24. Need more time for communication. I have questions about labour and give birth and want more communication. (P20, Chinese)*
	Language barriers	*25. Not always an interpreter was present. When a face to face interpreter were available (they) were used. However (the healthcare providers did) not always offer telephone interpreter. (P24, Korean)*

##### 3.2.1.2 Personal interpretation

Many women from CALD backgrounds expressed concerns about developing type 2 diabetes after pregnancy, but none of the Australian-born women had this concern (Table 2, Q4). Both CALD-background and Australian-born women were anxious and worried about their baby developing type 2 diabetes later in life (Table 2, Q5). In terms of risk factors for developing GDM, most participants from all ethnic groups mentioned age, family history, lack of physical activity, and poor dietary habits (Table 2, Q6). Two Chinese and one Indian participant also attributed their diagnosis to their ethnicity.

#### 3.2.2 Management issues

Many women from CALD backgrounds found dietary modifications challenging (Table 2, Q7). They reported reducing or avoiding consumption of cultural foods, opting for a more Western diet. Occasionally, they would feel hungry after meals but were afraid to eat more lest it would affect their blood glucose levels. In comparison, only one Australian-born woman was anxious about eating too much (Table 2, Q8). Two Chinese women commented that it was difficult to adhere to dietary restrictions in family gatherings (Table 2, Q9). One Chinese participant even stopped attending them altogether.

More women from CALD backgrounds than Australian-born women had challenges in monitoring their glucose levels. Some reasons included difficulty in monitoring blood glucose levels (BGLs) regularly, forgetting to take the glucose monitoring machine when they leave the house, and being too busy with work (Table 2, Q10). Some women, particularly Korean women, felt pain when pricking fingers (Table 2, Q11). Women from CALD backgrounds were also more likely to worry about their BGL results, especially when they did not exercise or had some carbohydrates prior to testing (Table 2, Q12).

While most Australian-born women were satisfied with their physical activity levels, women from CALD backgrounds had mixed experiences. Several barriers to being physically active included having to care for their children, busy with work, feeling lazy, and the weather getting cooler (Table 2, Q13). Two women from CALD backgrounds mentioned that physical activity did not help with managing their BGLs (Table 2, Q14).

#### 3.2.3 Roles of friends and family

##### 3.2.3.1 Assistants in management

All participants had some form of support from friends and family. Spouses played an important role in dietary modifications, such as adapting their eating habits to accommodate for women (Table 2, Q15). Spouses also accompanied participants in physical activity, such as going for walks after meals. Many women from CALD backgrounds would reach out to family and friends overseas for emotional support (Table 2, Q16). While most participants from CALD background found their family supportive, one Korean woman stated that her family was strict with dietary modifications and physical activity routines.

##### 3.2.3.2 Need of support group

Women from CALD backgrounds expressed a need for a support group with those who were also diagnosed with GDM. With family and friends overseas, having a support group would provide an opportunity for them to meet new friends. Three women from CALD backgrounds commented that they would feel more comfortable talking to those who shared similar experiences with them (Table 2, Q17). Contrarily, none of the Australian-born participants expressed the need for a support group.

#### 3.2.4 Information access

##### 3.2.4.1 Dietary information

Participants stated that the dietary information lacked advice for culturally specific foods, such as ways to adjust portion sizes for chapatti, rice, and noodles (Table 2, Q18). Two Indian women and one Australian-born participant commented that there was a lack of guidelines in dietary modification for vegetarians.

##### 3.2.4.2 Online resources

While only two Australian-born women used online resources to obtain more information about GDM, it was common for women from CALD backgrounds to browse the Internet and look for information written in their own language, as well as search for diabetes-friendly recipes that were culturally appropriate for them (Table 2, Q19).

#### 3.2.5 Experience with healthcare services

##### 3.2.5.1 Support from healthcare providers

Experiences in interacting with the healthcare providers were overwhelmingly positive for all participants. They provided lots of information regarding dietary modifications, physical activity, and glucose monitoring (Table 2, Q20). They also reassured patients, followed up with them to monitor their progress, and were readily available should the participants need assistance (Table 2, Q21).

##### 3.2.5.2 Consultations

The experiences were similar between women from CALD and non-CALD backgrounds; they commented that they preferred seeing the same specialist, e.g., obstetricians, midwives, rather than a different one every time (Table 2, Q22). Long waiting times for consultations was a common concern. Participants had to wait around an hour upon arrival. Participants often felt annoyed as they had to pay extra for parking fees and could not go to work afterwards. Some women had to take a day off work (Table 2, Q23). Furthermore, consultation times were too short and rushed. Participants spent approximately 10 min with the specialist. They would like more time to communicate with health providers so that their questions could be addressed (Table 2, Q24).

##### 3.2.5.3 Language barriers

Language barriers between the participant and healthcare providers were unique to women from CALD backgrounds and rendered participants feeling confused throughout the consultation, and the information from antenatal clinics was only provided in English. Undoubtedly, participants from CALD backgrounds found it easier to understand the information given to them when either the HCP spoke their language or when an interpreter was present. However, while it was encouraged to ask for an interpreter if required, interpreters were not always available, either in-person or via the telephone (Table 2, Q25).

## 4 Discussion

This study explored the experiences of GDM among women from CALD backgrounds compared to Australian-born women. 5 themes were identified: (1) reaction to diagnosis; (2) management issues; (3) roles of friends and family; (4) information access; and (5) experience with healthcare services. The results were in line with our hypotheses. Women from CALD backgrounds were more likely to have greater emotional responses and experienced more challenges in managing GDM.

### 4.1 Reaction to diagnosis

Feeling surprised upon diagnosis was universal among participants, but distress was more common among women from CALD backgrounds. Previous studies have reported similar marked emotional responses in women from CALD backgrounds with GDM (10, 16–18). Anxiety could be related to concerns about infant and maternal complications, such as the development of type 2 diabetes (17). As mentioned by participants in our study, HCPs, friends and family were important sources of emotional support, playing a crucial role in alleviating fear and anxiety associated with GDM (19). This suggested that health systems should utilize the women’s social network to enhance their emotional support.

In our study, most women from CALD backgrounds were aware of the aetiologias associated with GDM, such as ethnicity and family history of diabetes (7, 8, 20). In contrast, other studies demonstrated that women from CALD backgrounds had less appreciation of GDM due to poor language skills and health literacy (21, 22). Participants in this study might have gained knowledge about GDM in the educational sessions facilitated by diabetes educational nurses before the interviews. This could also be attributed to their high educational levels, with almost all of the participating women having a Bachelor’s degree or above. Interestingly, the emotional responses of women seemed to be independent of the knowledge about GDM. Support structures should prioritize providing emotional support to the diagnosis.

### 4.2 Management issues

One of the main challenges for managing GDM was a lack of culturally specific advice for dietary adjustments, in keeping with evidence from previous studies (18, 23, 24). Surprisingly, even Australian-born women adhering to specific diets, such as vegetarian, found that the dietary advice for managing GDM was inadequate. Restricting the consumption of cultural foods and the fear of eating were also reported in other studies (18, 23–25). Better education about food cultures and diets among health professionals can provide more tailored dietary information to women of all ethnicities.

Regarding glucose monitoring and physical activity, women from CALD backgrounds seemed to have worse experiences with more challenges than Australian-born women. This could be due to poor transition of knowledge to patients, which would lead to misunderstandings and maladaptive behaviours (12). Thus, information regarding lifestyle modifications should be more culturally tailored to enhance patients’ ability of self-management in GDM.

### 4.3 Roles of friends and family

Involvement of family and friends played an integral part in the management of GDM, particularly among women from CALD backgrounds (10, 26). Spouses were the main support for participants in dietary modification and physical activity (10, 12, 16). Due to the lack of support in Australia, women from CALD backgrounds would also reach out to friends and family overseas. A support group for these women can enhance both emotional and social support for women from CALD backgrounds.

### 4.4 Information access

Providing information and education about treatment options and prognosis can facilitate patient autonomy and self-care (19). The lack of culturally specific information on diet for ethnically diverse women is well established (12, 16, 23). In addition to the lack of culturally specific dietary information, another main finding related to this theme was that the use of online resources to obtain information about GDM was more common among women from CALD backgrounds than Australian-born women (27). The main reasons were to seek emotional support and share their experiences with other women (27, 28). Thus, health systems could utilize online resources to provide more information for women with GDM. For instance, patients could be referred to info graphs and short videos from the National Diabetes Service Scheme (NDSS) from Diabetes Australia, which are translated into various languages, including Chinese, Hindi, and Urdu (29).

### 4.5 Experience with healthcare professionals

The issues of long waiting times and short consultation times experienced by women from CALD and non-CALD backgrounds were described in another study (10). Language barrier was another challenge that women from CALD backgrounds faced. Korean women had more difficulty communicating with HCPs due to poor language skills. These factors would hinder their understanding of GDM, thus leading to poorer management (12, 21, 22). Providing information in various languages and improve the availability of interpreters would improve patients’ understanding of GDM (12).

### 4.6 Strengths and limitations

The study captured a comprehensive framework of women’s experience with managing GDM across various ethnicities. This was the first study to assess the experience of women with GDM born from China, India, Korea, and Australia.

There were several limitations to the study. First, while Chinese, Indian and Korean were the 3 most common ethnic groups in NSLHD, their experiences were not representative of all ethnic groups. Second, the impact of cultural festivals or practices, e.g., Ramadan, on the experiences of women during their pregnancy was not well captured. That is, women’s experiences of the same ethnic background could be different depending on when the study was conducted. Third, the sample size for Koreans were small compared to other ethnic groups. This could be associated with language barriers, which was particularly challenging for Korean participants. Finally, participants were recruited within the geographical boundaries of Northern Sydney, which might not be characteristic of other regions in Australia.

## 5 Conclusion

Women from CALD backgrounds experienced unique challenges in managing GDM. They were more likely to feel distressed, have difficulty adjusting their diet, lack social support, poorer access to information regarding GDM management, and face more challenges when interacting with healthcare providers. Models of care for women with GDM should be better tailored to various ethnic backgrounds, such as providing more culturally tailored dietary information, ensuring the availability of interpreters, and improving language diversity in the information provided to patients. Healthcare systems could also enhance the social support for women with GDM by involving friends and family in diabetes education and organizing peer support groups for women with GDM. Antenatal care clinics could also utilize online resources to distribute information to all women with GDM for better accessibility.

## Data availability statement

The raw data supporting the conclusions of this article will be made available by the authors, without undue reservation.

## Ethics statement

The studies involving humans were approved by This project was approved by the Northern Sydney Local Health District Human Research Ethics Committee on 18 November 2016 (Reference No.: LNR/16/HAWKE/417). The studies were conducted in accordance with the local legislation and institutional requirements. The participants provided their written informed consent to participate in this study.

## Author contributions

HL: Formal analysis, Writing – original draft. JD: Writing – review & editing. TW: Writing – review & editing. MC: Methodology, Writing – review & editing. LO: Writing – review & editing. GF: Writing – review & editing. SG: Supervision, Writing – review & editing.
